# Competition and cooperation in a synchronous bushcricket chorus

**DOI:** 10.1098/rsos.140167

**Published:** 2014-10-08

**Authors:** M. Hartbauer, L. Haitzinger, M. Kainz, H. Römer

**Affiliations:** Institute of Zoology, Karl-Franzens University Graz, Universitätsplatz 2, Graz 8010, Austria

**Keywords:** chorus evolution, cooperation, beacon effect, sexual selection, leader preference, rhythm preservation

## Abstract

Synchronous signalling within choruses of the same species either emerges from cooperation or competition. In our study on the katydid *Mecopoda elongata*, we aim to identify mechanisms driving evolution towards synchrony. The increase of signal amplitude owing to synchronous signalling and the preservation of a conspecific signal period may represent cooperative mechanisms, whereas chorus synchrony may also result from the preference of females for leading signals and the resulting competition for the leader role. We recorded the timing of signals and the resulting communal signal amplitudes in small choruses and performed female choice experiments to identify such mechanisms. Males frequently timed their signals either as leader or follower with an average time lag of about 70 ms. Females selected males in such choruses on the basis of signal order and signal duration. Two-choice experiments revealed a time lag of only 70 ms to bias mate choice in favour of the leader. Furthermore, a song model with a conspecific signal period of 2 s was more attractive than a song model with an irregular or longer and shorter than average signal period. Owing to a high degree of overlap and plasticity of signals produced in ‘four male choruses’, peak and root mean square amplitudes increased by about 7 dB relative to lone singers. Modelling active space of synchronous males and solo singing males revealed a strongly increased broadcast area of synchronous signallers, but a slightly reduced *per capita* mating possibility compared with lone singers. These results suggest a strong leader preference of females as the ultimate causation of inter-male competition for timing signals as leader. The emerging synchrony increases the amplitude of signals produced in a chorus and has the potential to compensate a reduction of mating advantage in a chorus. We discuss a possible fitness benefit of males gained through a beacon effect and the possibility that signalling as follower is stabilized via natural selection.

## 2. Introduction

Insects and anurans often aggregate at common feeding and/or breeding sites, where simultaneously active signallers form an acoustic lek termed spree [1–3]. The evolution of such aggregates can also be the consequence of sexual selection as females may prefer calling songs produced in a group over songs of lone singers (e.g. [4–6]). As a consequence, males often show a phonotactic response to calls of competitors broadcast at medium intensity [7]. In some species, signals generated in a group are precisely timed relative to those of others, leading to fascinating group displays. One of the most spectacular forms of long-range sexual signalling can be found in tropical fireflies where thousands of males aggregate and produce rhythmic flashes in synchrony [8–10]. Buck [11] discussed the selective forces driving the evolution of firefly synchrony, and Greenfield [12] suggested analogy in the evolution of chorus synchrony in acoustically communicating insects.

Greenfield [13,14] reviewed various hypotheses explaining the evolution of synchronous mating displays in insects and anurans. One reason for timing signals in synchrony is the increase in peak signal amplitude, termed ‘beacon effect’ by Buck & Buck [8,15], similar to the ‘signal enhancement hypothesis’ [16,17]. As the result, the signal-to-noise-ratio (SNR) and the active range of the synchronous group signal is increased, and receivers detect the group signal at greater distances compared with individuals singing in isolation. This hypothesis suggests chorus synchrony as the outcome of inter-male cooperation. However, the improvement of SNRs is only relevant if receivers evaluate peak signal amplitude instead of integrating signals over a longer time span [14]. Furthermore, a true advantage for cooperating males to produce the ‘beacon effect’ is only given if the *per capita* mating advantage is higher compared with single signallers.

An alternative hypothesis postulates chorus synchrony as the outcome of a collective effort of males aiming to preserve a signal rhythm that is attractive for females (e.g. *Oecanthus fultoni* [18]). This seems to hold true for *Neoconocephalus nebrascensis* where synchronously interacting males separate subsequent verses by a minimum amplitude modulation of 20 dB in order to keep communal songs attractive for females [19]. In some acoustically communicating insect species, males benefit from synchronous signalling through improved perception of female acoustic responses within silent intervals [13]. Similarly, flash synchrony of *Photinus carolinus* males reduces visual clutter and increases the likelihood of detecting a female flash response [20].

Chorus synchrony may also be the outcome of competition among signallers. Females of many insect and anuran species prefer signals slightly timed in advance to the signals of competitors, which forces males to compete for timing their signals as leader. In this way, chorus synchrony emerges as a by-product of competition (fireflies: [20,21]; anurans: [22]; katydids: e.g. [23–28]). As males joining a chorus expose themselves to higher inter-male competition, it is likely that they gain fitness benefits that outweigh increased competition. Unfortunately, it is often difficult to disentangle female mating preferences, inter-male competition and benefits gained through synchronous signalling in groups, such as a lower *per capita* risk of predation for signallers joining a group [29–31].

Imperfect chorus synchrony has been known for the katydid *Mecopoda elongata* (Orthoptera: Tettigoniidae) since 1990 [32]. The timing of the male signals in imperfect synchrony has fundamental consequences for a male's attractiveness because females prefer leading signals over identical signals broadcast with a time lag of 140 ms [26]. However, different from other synchronizing insects such as *Neoconocephalus spiza* [25], *M. elongata* males establish persistent leader and follower roles in duets [33], which questions chorus synchrony as the outcome of inter-male competition for timing signals as leader. In our study, we investigate whether chorus synchrony in this katydid is either the outcome of competition or cooperation, whereby the latter may lead to an increase in the amplitude of communal signals. We studied the precision of signal timing in small male choruses, its effect on peak signal amplitude and female mate choice. In a modelling approach, we simulated the broadcast area of synchronous signallers to estimate the consequence of a ‘beacon effect’ regarding mating possibilities of individual chorus members. In order to reveal a possible ultimate mechanism that stabilizes the species-specific signal period of about 2 s, *M. elongata* females were given the possibility to select among calling song models exhibiting different signal periods.

## 3. Material and methods

### 3.1 Animals

All experiments were performed with the tropical katydid species *M. elongata*, named species ‘S’ by Sismondo [32]. These insects were originally collected in the field in Malaysia and reared at the Department of Zoology at the Karl-Franzens University of Graz. Ambient temperature and relative humidity in the breeding room were maintained at 27°C and 70%, respectively. The light : dark regime followed a 12 : 12 h schedule. Males usually start singing right after the onset of the dark phase. Insects were fed ad libitum with water or water gel, oat flakes, fish food and lettuce. Individuals were identified using a two-colour dot code on their pronotum. Females participating in choice experiments were spatially, but not acoustically separated from males after completing their final moult.

### 3.2 Experiment 1: song recordings in small choruses

Acoustic interactions of males were studied in 18 choruses, each consisting of four randomly selected males. Males were positioned in a square with a minimum distance of 2 m separating neighbouring males. Males were caged in cylindrical tubes (diameter: 7 cm, height: 24 cm) made of wire mesh. Insect cages were placed in an upright position on cubic wire mesh boxes to elevate males at least 25 cm above ground. Singing of each male was recorded with a separate microphone positioned next to a caged male (Voltcraft Inc. sound level meter). The insensitive setting of these microphones allowed recording of the song of only the nearest male. Microphone signals were digitized using an A/D converter (Power 1401 mk 2, Cambridge Electronic Design Limited, Cambridge, UK) operating at a sampling rate of 10 kHz. A further microphone with a flat frequency response up to 40 kHz was used to record the additive effect of the four signals in the centre of the chorus (1/2′, type 40AC, serial no. 80264; 1/2′ preamplifier type 26AM, serial no. 86313; G.R.A.S. Sound & Vibration, Denmark) which results in an male–microphone distance of 1.4 m. The output of this centre microphone was amplified using a power module (type 12AK, serial no. 69498; G.R.A.S Sound & Vibration, Denmark). A/D conversion of centre microphone signals was achieved with the Power 1401 operating at a sampling rate of 83 kHz. The centre microphone was in the same horizontal plane as the singing insects (30 cm above ground). As a result of the directionality of this microphone and its vertical orientation, signal components with a carrier frequency higher than 8 kHz were attenuated in their amplitude; attenuation increased linearly with carrier frequency (8 kHz: −3 dB; 20 kHz signals—10 dB; 30 kHz: −15 dB). The resulting low-pass filter effect roughly simulates the excess attenuation of high-frequency and ultrasonic signal components in the natural habitat of katydids [34,35]. All chorus recordings were performed in an acoustic chamber (M:Box, Desone Modulare Akustik, Berlin, Germany; dimension 280×220×200 cm), with the walls inside this chamber covered with sound absorbing foam exhibiting a wedge length of 3 cm. Sound recordings were restricted to the first 4 h of the dark cycle. Ambient temperature and humidity was controlled using a fan heater (DeLonghi, Italy) and a humidifier. Temperature and relative humidity in the sound chamber was on average 26±1.5°C and 60%, respectively.

A custom-written script was developed to evaluate the temporal relationship between all five microphone signals (v. 5.21, Spike2, Cambridge Electronic Design Limited). After high-pass filtering (cut-off frequency 500 Hz) to remove low-frequency noise, a manual threshold in each microphone channel was set, which allowed us to automatically determine the time of signal onset and signal duration. Another Spike2 script was used to evaluate the peak as well as root mean square (RMS) amplitude of communal signals recorded in the centre of the chorus. Evaluation of signal amplitudes was restricted to sequences of recordings in which chorus attendance of all four males remained rather constant. The communal signal amplitude of males interacting in a chorus is given in dB values relative to the signal of solo singing males contributing to choruses in the same setting and same song bout. This method assumes that males neither change their singing position nor their loudness when they sing in a solo setting or in a chorus with other males. Relative dB values were averaged across 40–220 subsequent signal interactions or solo singing chirps.

For a comparison of the signal period of males joining a chorus with their intrinsic chirp period (CP), the songs of the same males singing in isolation were recorded inside a temperature-controlled incubator at a constant temperature of 27±1°C within 1–2 days before or after the chorus experiment. The intrinsic signal period of a male was determined by averaging signal period over 40 subsequent chirps that were produced at the end of the first third of the song bout.

### 3.3 Experiment 2

In a first set of two-choice experiments, we studied female preference for song models differing in relative signal timing, absolute signal period or signal period variability. Females were released at a distance of 210 cm from two speakers positioned in the rear of the arena. Speakers were separated by 155 cm and elevated 9 cm above a soft carpet covering the floor of the arena. Prior to each experiment, females were placed in wire mesh tubes (diameter: 7 cm, height: 24 cm) in an incubator at a constant temperature and relative humidity of 25°C and 70%, respectively. Females were allowed to adapt to the experimental conditions for about 5 min at the release point. Then, the plug of the cage was manually removed and acoustic stimulation was turned on. Trials were regarded as positive when the distance between females and the chosen speaker was less than 30 cm. If a female remained inside its cage, or left the cage without approaching a speaker within 15 min, the trial was aborted and treated as negative. All trials were conducted in complete darkness. Phonotactic paths were recorded with an infrared-camera (GKB CB-38075) and infrared illumination (EuroTECH LED, 850 nm) mounted at the ceiling of the arena. For later analysis, pictures of the arena were taken at intervals of 3–5 s. The air temperature and relative humidity was controlled by an air humidifier and a fan heater. The average ambient temperature during phonotaxis experiments was about 26°C and relative humidity was on average 53%.

Individual females were tested up to four times in the first 7 h of the dark cycle. Subsequent trials performed with the same individual were interrupted by pauses lasting for at least 2 h. Song models with different signal periods were randomly assigned to each female in order to prevent a bias due to the trial sequence. Each stimulus pair was switched between both speakers to prevent a bias arising from a possible handedness of females or the spatial details of the arena. Song model pairs were presented for as many times as necessary in different trials (performed in different nights) to end up with four positive trials per female (except songs with a CP of 1.0 s). This kind of data replication was considered in the statistical evaluation (see below). A total of 12–15 females contributed to positive phonotaxis trials.

### 3.4 Experiment 3

Females were given a choice between two song models; one is based on a signal that was recorded in the centre of a small chorus and the other consists of a chirp of a solo singing male. Two loudspeakers were positioned at opposite corners of the arena. The song of a solo singing male and the signal recorded in a small chorus consisting of four males was broadcast simultaneously (see the electronic supplementary material, figure S1). In this experiment, the amplitude of the chorus signal was increased by 7 dB compared with the solo song, mimicking the beacon effect resulting from signal overlap of synchronously singing males (see Results). This was achieved by reducing the attenuation of the chorus signal by 7 dB after calibrating competing signals to 60 dB sound pressure level (SPL). Calibration is based on the continuous broadcast of the three final syllables with the highest amplitude in the solo song or the continuous broadcast of an equivalent section of the chorus signal. Calibration was performed with a CEL 414 sound level meter (Casella, Bedford, UK) operating in slow integration mode (RMS) by using a 1/2′ microphone (type 2540, serial: 1898, Larson Davis, Depew, NY, USA). Females were released exactly midway of both speakers separated by 316 cm. Again, trials were regarded as positive when the distance between females and the chosen speaker was less than 30 cm.

### 3.5 Experiment 4

In a third set of choice experiments, females were released in the middle of a small chorus consisting of four synchronously interacting males. Males were caged and arranged in a square; the distance between neighbouring males was 200 cm. A microphone (Voltcraft Inc., Germany) was placed next to each male to record the sound of each male separately. The microphone output was digitized through four channels of the A/D converter (Power 1401 mk2, Cambridge Electronic Design Limited). The release of the female started as soon as four males were singing in synchrony. The experiment was aborted and the corresponding male was regarded as selected when a female entered a circle (invisible for females) with a radius of 30 cm surrounding each male. Thirteen females were tested in 10 different choruses. Signals recorded during mate choice were analysed with a custom-written Spike2 script to identify the timing and duration of signals.

### 3.6 Song models and playback

**Figure 1.**
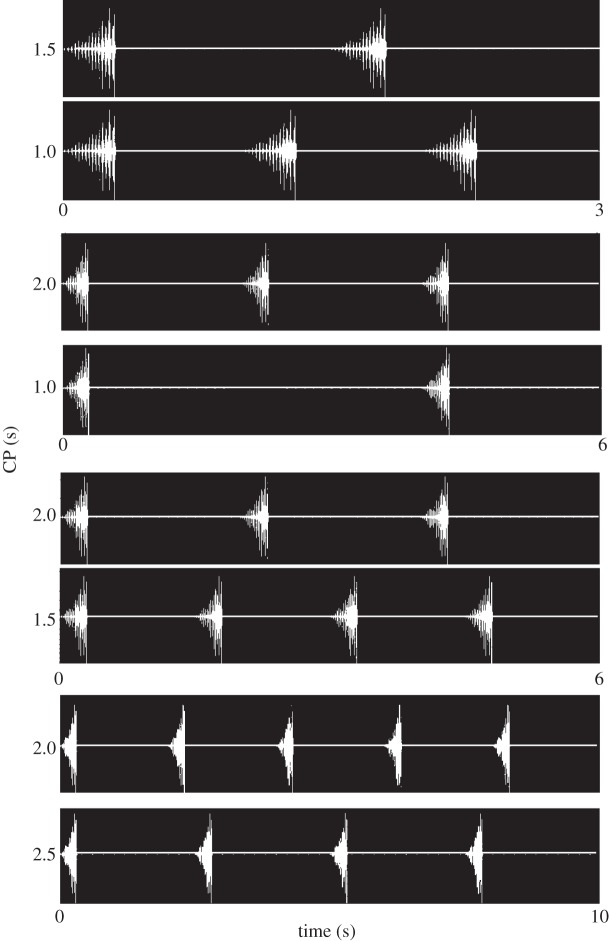
Playback signals used in two-choice experiments. Song models with different signal periods used in two-choice experiments. Note that a partial overlap of chirps is absent in simultaneously broadcast song models.

A representative chirp of a solo singing male was used to synthesize model songs with different CPs using the audio editing software Cool Edit Pro (Syntrillium Inc.). The chirp consisted of 16 syllables increasing in amplitude (total signal duration 308 ms) and was recorded at a distance of 15 cm from a singing male using a 1/2′ microphone (type 40AC, G.R.A.S). The natural CP is about 2 s at an ambient temperature of 27°C [33]. The relative signal timing of chirps in simultaneously broadcast song models is illustrated in figure 1. The following CP combinations were tested: 1.0 versus 1.5 s, 1.5 versus 2.0 s, 2.0 versus 2.5 s, 2.0 versus 1.0 s, 2.0 s versus random CP. As a strong leader preference may override a possible preference for a certain CP [26], consistent leader–follower situations were avoided during acoustic playbacks (figure 1). CP in the ‘random song model’ varied randomly between a minimum of 1 s and a maximum of 3 s (the average CP was exactly 2 s). The song model lasted for 2 min and was broadcast in loop mode using Cool Edit Pro. The influence of relative signal timing on female choice was investigated by broadcasting identical chirps through separate speakers with a fixed time lag of either 70 or 140 ms at a signal period of 2.0 s.

An external multi-channel soundcard (type FA-101, Edirol, Roland Corporation, Japan) controlled via Cool Edit Pro (Syntrillium Inc.) was used for the playback of sound signals. Playback signals were attenuated in steps of 1 dB using a programmable signal attenuator (PA5, Tucker-Davis technologies, FL, USA) connected to a high-end stereo power amplifier (C272, NAD Electronics International, Ontario, Canada). Signals were broadcast through string tweeters with almost identical frequency response properties (LEAF Tweeter Technics EAS- 10TH400A, Kadoma, Japan). Sound calibration was performed while three loud syllables of chirp signals were broadcast in loop mode. Signals were calibrated to 70 dB SPL at the release site of females using a CEL 414 sound level meter (microphone: type 2540, Larson Davis, Depew, NY, USA, serial: 1898) operating in slow integration mode. In the second set of choice experiments, the solo chirp was calibrated to a SPL of 60 dB and the ‘chorus signal’ to 67 dB using the same method. A higher SPL in the first set of choice experiments was chosen to simulate a female that had already entered a chorus.

### 3.7 Modelling the active space of a signal

The area in which receivers are able to detect a signal is defined as the ‘broadcast area’ [36] or ‘active space’ [37]. As a beacon effect leads to an increase of this area (see Results), a chorus of synchronously signalling males is expected to attract a higher number of females compared with single singing males. We simulated the active space of synchronous signallers by means of a two-dimensional multi-agent computer model that was developed in Netlogo (v. 5.0). Simulations of the active space are based on realistic sound propagation properties observed in the field [35] and take into account the hearing threshold of receivers, which was set to either 40 or 50 dB SPL, whereby a hearing threshold of 40 dB SPL roughly corresponds to silent laboratory conditions [38,39]. Mecopoda choruses have been recorded in the field exclusively in rainforest clearings where variation in the third dimension was small (M. Hartbauer 2012, personal observation). We therefore decided to restrict simulations to only two dimensions. SPL of signallers was set to 81 dB, a value that corresponds to the average peak SPL of *M. elongata* males measured at a distance of 1 m (*N*=7). Signal attenuation over distance was simulated for a pure tone with a carrier frequency of 10 kHz signal and was modelled after equation (2.1), which simulates a realistic excess attenuation of this signal [35]. Attenuation of acoustic signals depends on the carrier frequency and on the position of the signaller relative to ground. The carrier frequency of 10 kHz was chosen because auditory neurons of *Mecopoda* are tuned to carrier frequencies higher than 10 kHz [38–40]. The additive effect of several signals of synchronous signallers was simulated for up to four signallers arranged in a square. Simulations were performed with nearest neighbour distances of 2, 5 and 10 m. Summation of incoherent sound sources was calculated after equation (2.2) and leads to an increase of 6 dB if four identical signallers are active at the same time:

3.1 $$\begin{array}{rl} I & =81\;\text{dB}-(14.18\times \text{ln}(x)-0.981), \end{array}$$

3.2 $$\begin{array}{rl} \text{sum intensity} & =10\times (\log((10^{\left(\mathrm{sum}\;\mathrm{intensity}/10\right)})+(10^{\left(I/10\right)}))), \end{array}$$

where *I* denotes the SPL of a signaller measured at a distance *x*.

The active space of signallers correlates with the number of attracted females if one assumes a homogeneous distribution of females. In this modelling approach, we simulated different densities of females (one female/33 m^2^; one female/100 m^2^; one female/150 m^2^) as there are no data available about real female abundance. The number of females theoretically attracted to a chorus was calculated by dividing the simulated active space of the communal signal (given in m^2^) by the m^2^ in which on average one female occurs. Per capita mating possibilities of males was calculated by dividing the number of attracted females by chorus size.

### 3.8 Statistical analysis

With the exception of data gathered in female choice experiments, all statistical analyses were performed in Sigmaplot (v. 12.0). Datasets were checked for linearity before performing parametric tests. A significant preference of females for a song model in two-choice situations was investigated in RStudio (v. 096.230, R v. 2.13.1) by application of a generalized binomial mixed model (GBMM) that was fitted by Laplace approximation. In this model, female identification acted as a random intercept. The Raleigh test was performed in RStudio using the circular statistics package.

## 4. Results

### 4.1 Experiment 1

#### 4.1.1 Acoustic interaction of males in a chorus

**Figure 2.**
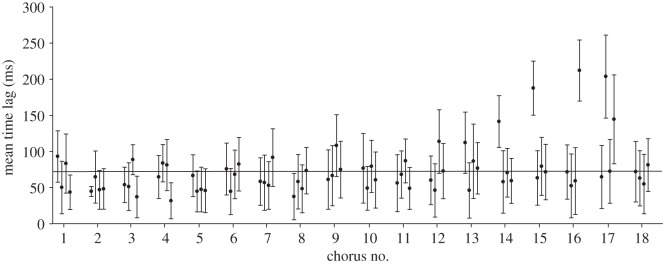
Signal timing in choruses. Mean time delay between signals produced by individuals in different choruses. The horizontal line indicates the averaged time delay obtained in 18 choruses.

As members of a ‘four male chorus’, males preferably sing when other males are active at the same time. Evaluation of chorus attendance in experiment 1 revealed that males spent a significantly higher proportion of time singing together with other males compared with solo singing episodes (*p*<0.05, ANOVA on ranks). Fine temporal analysis of signal timing of four simultaneously active males revealed a high degree of signal overlap, which is the consequence of a rather small average time difference between the onset of leading and follower signals (73±34 ms, mean ± s.d. of 18 choruses; indicated by a horizontal line in figure 2). An average time lag of 73±34 ms corresponds to a phase lag of 14.5±3.5° of three follower males relative to the signal period of the leader and significantly deviates from random signal timing (*p*<0.001, Raleigh test). In only four out of 18 choruses, some males exhibited a time lag of more than 150 ms, corresponding to a phase lag of 29.8° (see chorus 14–17 in figure 2). Evaluation of individual chirp timing in ‘four male choruses’ showed that in 68% of all choruses, a single male attained leadership in more than 50% of periodic signal interactions (occurring on average every 2 s). In 11 choruses, males interacted in multiple song bouts (lasting for a minimum of 10 min), whereby in 64% of these choruses the same individual attained frequent leadership in at least two subsequent song bouts. Furthermore, 71% of males maintained their leader/follower roles in at least two different choruses where they interacted with different males.

#### 4.1.2 Signal period in a chorus

**Figure 3.**
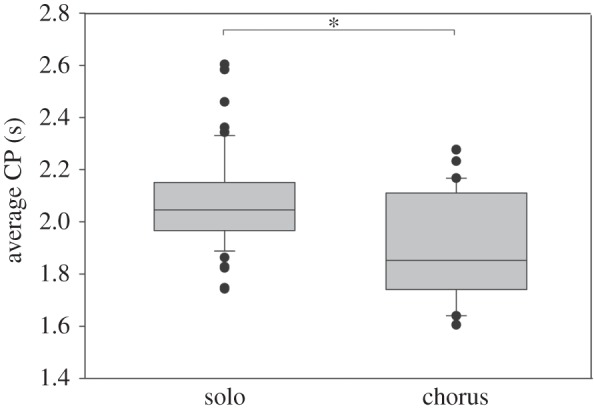
CPs of solo singing males and males attending a chorus. Average CP of males singing in isolation and males attending a chorus. *p<0001; paired *t*-test, *N*=70 (53 males).

Evaluation of the signal period of males either singing in isolation or together with three other males in a chorus revealed a significantly shorter average CP of males joining a chorus (chorus 1.88 s; solo 2.09 s; paired *t*-test, *p*<0.001, 53 males; figure 3). This is true for both leader and follower males, which reduced their CP compared with the solo singing situation by 4% and 7%, respectively (electronic supplementary material, figure S2). The difference between the change of CPs of leader and follower males was not significant (*p*>0.05, Mann–Whitney rank sum test). In four out of 11 choruses, males timing their chirps as leader in at least 50% of signal interactions exhibited the shortest intrinsic CP compared with other chorus participants. In the remaining choruses, leadership was not dominated by a single male or the male dominating leadership exhibiting a slightly slower intrinsic signal rate compared with competitors.

#### 4.1.3 Beacon effect

**Figure 4.**
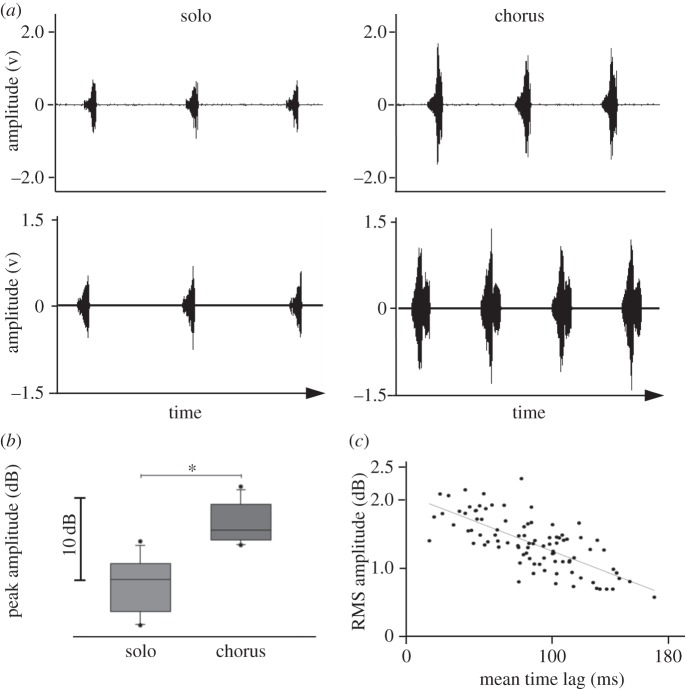
Amplitude of communal signals. (*a*) Examples of signals recorded in the centre of a chorus when only one male was active (left) or four males were singing synchronously (right). The upper panel shows the signals recorded within a chorus with a higher degree of signal overlap compared to the chorus shown below. (*b*) Average relative peak signal amplitude of solo singing males and four males signalling in synchrony (*p*<0.001, paired *t*-test, *N*=12). (*c*) Correlation between the average time difference of chirps produced in a chorus (one song bout) and corresponding RMS signal amplitudes (dB values of chirps are shown relative to the smallest RMS amplitude obtained during this song bout). (*N*=93; *R*^2^=0.52).

**Figure 5.**
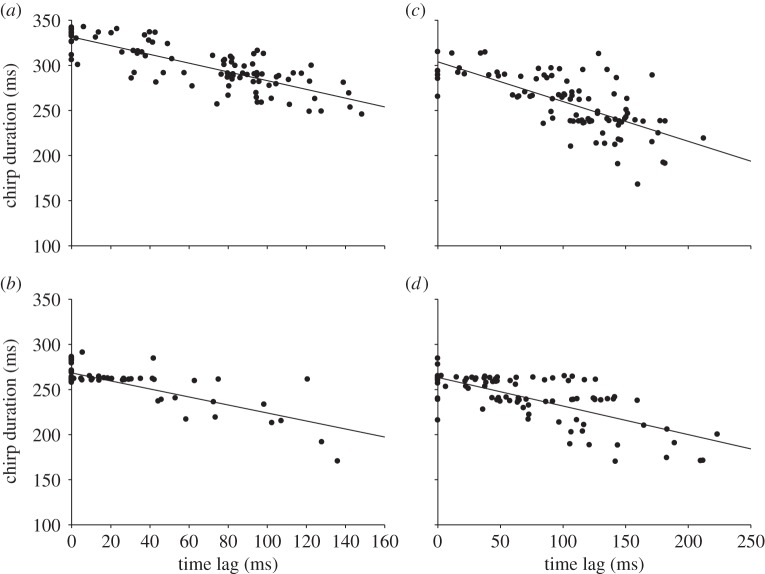
Plasticity of signals generated in a chorus. Correlation between signal timing and signal duration of four males (*a*–*d*) belonging to a synchronous chorus. Data shown were obtained from one song bout. Each dot represents the relative signal timing and signal duration of a chirp produced by a male. Spearman's rank-order correlation coefficients: (*a*), −0.77; (*b*), −0.38; (*c*), −0.69; (*d*), −0.62 (*p*<0.0001).

Not all males in a chorus started and ended singing at the same time, which allowed for the comparison of the signal amplitude of solo singing males with the additive amplitude effect of four synchronously singing males in experiment 1. Figure 4*a* shows two examples of microphone recordings obtained in the chorus centre when either four males were active at the same time or one male was singing. A high degree of signal overlap in the example shown in the top panel leads to an increase of RMS signal amplitude by 6.9 dB (maximum signal amplitude increase 7.9 dB), whereas a low degree of signal overlap in another chorus (bottom panel) raised RMS amplitude by only 2.7 dB (maximum signal amplitude increase 4.1 dB). Synchronous signalling resulted in an average increase of the maximum signal amplitude by 7.3±3.5 dB (RMS amplitude increase 7.5±2.3 dB) relative to the amplitude of solo singing males (*p*<0.001, *N*=12, paired *t*-test; figure 4*b*). The average degree of signal overlap in choruses of four males was high, which is obvious in a rather moderate increase of 47% in the duration of chorus signals relative to solo signals (chorus: 384±95 ms, solo: 262±26 ms). The tight correlation between signal amplitude and signal timing in a ‘four male chorus’ is shown in figure 4*c*. In the case of small time differences between males, RMS amplitude of chorus signals increases by about 2 dB relative to the minimum values observed in this chorus situation where four males were singing simultaneously. However, as time differences between signals increased, RMS amplitude decreased. A similar decrease of signal amplitude with increasing time differences was found in 11 out of a total of 12 ‘four male choruses’ (*p*<0.001, average correlation coefficient: −0.48, Spearman's rank-order correlation). This result seems to contradict the average increase in signal amplitude by 7.3 dB reported above, but in the example shown in figure 4*c* four males were permanently active and only the degree of signal overlap varied. Therefore, the result only demonstrates the effect of the degree of overlap, not the increase in SPL as the result of additional signals. Interestingly, males in all choruses gradually decreased the duration of their signals as the time difference between leading and follower signals increased (see significant negative correlation coefficients in the example of a chorus shown in figure 5).

We also studied the effect of a ‘four male chorus’ on signal amplitude using four loudspeakers substituting real males. Each of these speakers broadcast identical chirps with an RMS amplitude of 60 dB (corresponding to a peak signal amplitude of 71 dB SPL) equidistant to a microphone positioned in the centre of the speaker arrangement. Sound level measurements showed that synchronous playback of chirp signals led to an average increase of peak signal amplitude by 7.5 dB (RMS amplitude=6.5 dB) relative to single speaker playbacks. When two of these speakers broadcast signals with a delay of 70 ms, peak signal amplitude increased by 4.5 dB (RMS=3.5 dB) relative to single speaker playbacks.

#### 4.1.4 Simulation of the active space of a chorus

**Figure 6.**
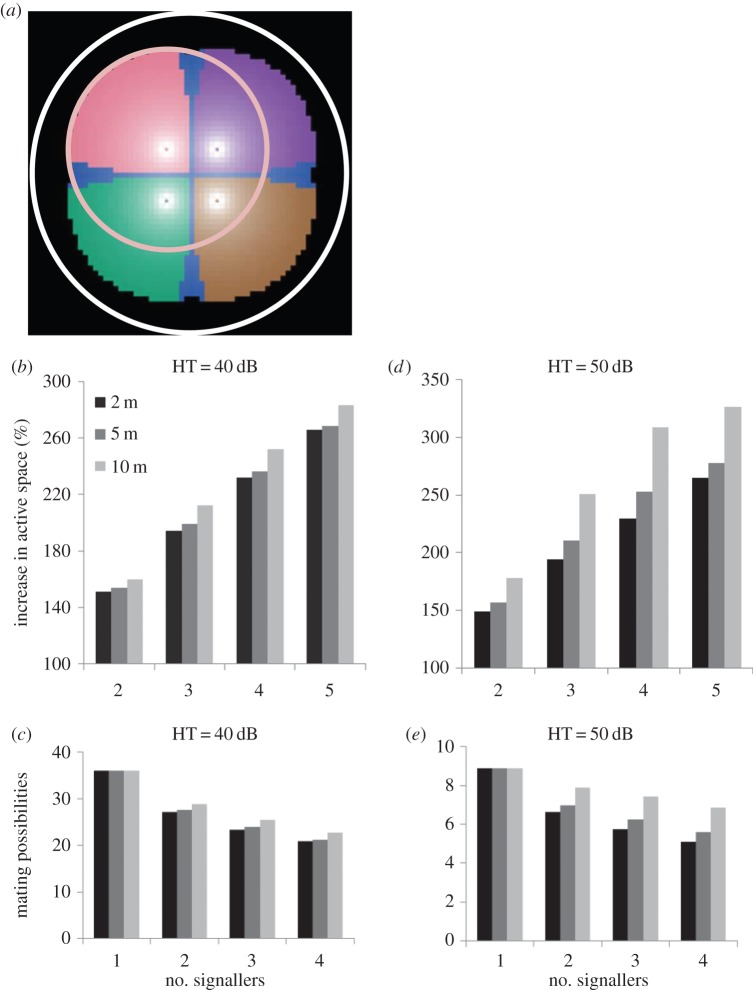
Computer simulation of the active space and females attracted to a chorus. (*a*) Simulation of the active space of a lone singing male (pink circle) and the active space of four males signalling in synchrony (white circle). Different colour-coded regions indicate the area in which individual signallers are at least 1 dB louder than competitors. (*b*,*d*) Increase in active space of simultaneously broadcast signals generated by two to five signallers relative to a lone signaller. Simulation runs were performed with a theoretical hearing threshold of 40 dB SPL (*b*) or 50 dB SPL (*d*). (*c*,*e*) Theoretical number of per capita mating possibilities of males signalling in synchrony assuming a female density of one individual per 33 m^2^ and a hearing threshold of either 40 dB SPL (*c*) or 50 dB SPL (*e*). Grey values in (*b*–*e*) indicate minimum inter-male distances, except for a five male chorus where one sender is located in the centre of four senders that are arranged in a square. For simulation details, see Material and methods.

The temporal overlap of signals in a chorus leads to an increase in the area in which chorus signals can be detected by receivers (active space). In order to study this in more detail, we simulated the active space of identical signallers emitting a pure tone signal with a carrier frequency of 10 kHz and a SPL of 81 dB assuming a hearing threshold of receivers of either 40 or 50 dB SPL. The active space of solo singing individuals is mainly influenced by the hearing threshold of receivers. By contrast, the active space of simulated males in a chorus is significantly reduced if signallers are within hearing range of each other because the area in which their signals are at least 1 dB louder than the signals of others is strongly restricted (compare the dimension of the pink circle with pink area in figure 6*a*). At the same time, however, chorus synchrony increases the active space of chorus signals with the number of signallers (figure 6*b*,*d*). The active space is only weakly affected by the minimum inter-male distance if the hearing threshold of receivers is set to 40 dB SPL (figure 6*b*). In this scenario, three or four signallers increase the active space 2.0 or 2.5 times relative to a single individual, respectively. Increasing the hearing threshold of receivers to 50 dB SPL leads to a strong reduction in the active space of individuals and inter-sender distance becomes an important parameter for the dimension of the active space (figure 6*d*). In this scenario, four signallers separated by 10 m increased their active space three times compared with a lone signaller, but at shorter inter-male distances the increase in active space with chorus size was similar compared with results obtained with a hearing threshold of 40 dB SPL. Simulating the active space of a solo singing male is comparable to the simulation of deaf males. Owing to the low duty cycle of loud syllables, accidental temporal overlap rarely occurs and the active space of deaf males in a chorus with other deaf males is equal to solo singing males at the time of signal production. Assuming a rather high density of females of about one individual per 33–100 m^2^ and a hearing threshold of 40 dB SPL increased the number of attracted females linearly with chorus size, but the average per capita mating possibility simultaneously decreased (figure 6*c*). This effect was weaker in simulations where the active space was reduced owing to a higher hearing threshold of 50 dB SPL (figure 6*e*).

### 4.2 Experiment 2

#### 4.2.1 Two-choice experiments

**Figure 7.**
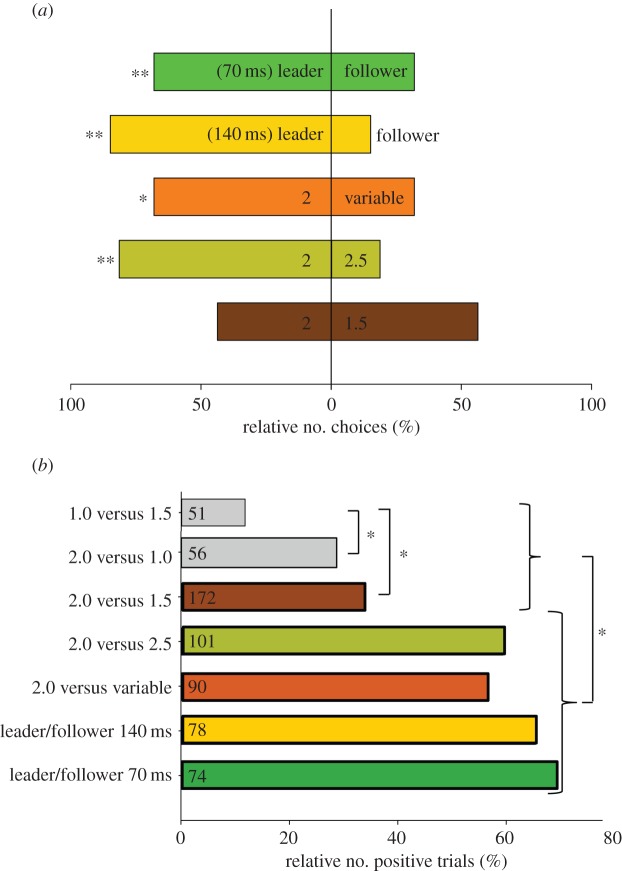
Female choice results. (*a*) Percentage of positive phonotaxis runs to either song model in different two-choice situations. ^**^p≤0.01, *p≤0.05. Data are based on 48 positive runs obtained from 12 individuals. See Material and methods for GBMM statistics. (*b*) Motivation of females to approach any speaker in various two-choice situations. Values represent the percentage of positive trials. Values inside bars represent the total number of trials. Grey bars indicate stimulus situations in which female motivation was too low to test for a significant preference. *p<0.01, *z*-test.

In 80% of positive trials, females showed a significant preference for the leading signal in experiment 2 when identical chirps were presented from two different directions with a time lag of 140 ms (*p*<0.01, GBMM; 12 females tested). A similar result (70%) was obtained when the lead was reduced to only 70 ms (*p*<0.01, GBMM; figure 7*a*).

When females were given the choice between a song model with a fixed CP of 2 s and a song exhibiting a variable CP, females approached song models with a fixed CP significantly more often (*p*<0.05, GBMM; figure 7*a*). Females also demonstrated a significant preference for the faster chirp rate in a choice situation between a CP of 2.0 and 2.5 s (*p*<0.01, GBMM). By contrast, in a choice between a CP of 2.0 and 1.5 s, the result was no significant preference for the faster CP. Apparently, the motivation of females to approach any speaker was very low in trials when song models with very short CPs of 1.5 or 1.0 s were used (figure 7*b*). Therefore, choice experiments in which short CPs of 1.5 or 1.0 s were broadcast to females did not provide sufficient data to perform statistical tests despite a rather high number of tested females.

### 4.3 Experiment 3

#### 4.3.1 Female choice between a solo and chorus signal

When females were given the choice between chirps of a solo singing male and the chorus signal in experiment 3, in 63% of trials females approached the chorus signal and in 37% of trials the solo song model. This was the case although the amplitude of the chorus model was 7 dB higher relative to the single chirp model. As each female was tested two times, there was no significant preference for the chorus song model in this choice situation (*p*=0.134, GBMM, *N*=38). In a control experiment, the chirp model of a single singing male was broadcast in synchrony from opposite directions with the same difference in signal amplitude (7 dB). In 72% of trials, females approached the loudspeaker broadcasting the louder song significantly more often (*p*<0.01, GBMM, *N*=29).

### 4.4 Experiment 4

#### 4.4.1 Female choice in a ‘four male chorus’

**Figure 8.**
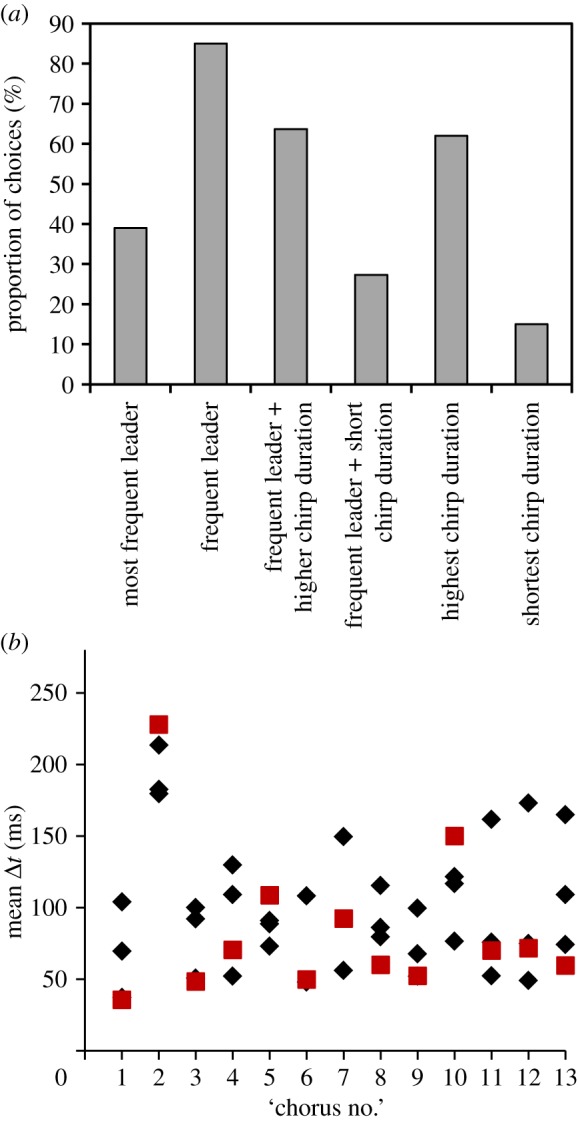
Female choice in small choruses. (*a*) Signal properties of males that were selected by females in different ‘four male choruses’ (13 females tested in 10 different choruses). Arbitrary choice among males corresponds to a 25% level. Axis legend: most frequent leader: male producing the highest number of leading signals in a chorus. Frequent leader: male producing most or second most leading signals selected by 11 females. Higher chirp duration: the average chirp duration of the selected male is similar (±15 ms) compared with the male exhibiting the highest mean chirp duration. Short chirp duration: the selected male has an average chirp duration that is similar (±15 ms) compared with the male with the shortest chirp duration. (*b*) Average time difference of signals produced by male individuals during mate choice. Red squares represent the average time difference of selected males.

Females released in the middle of a chorus consisting of four imperfectly synchronized males on average spent 293±99 s (about 146 subsequent signal interactions) to approach a male. In experiment 4, females selected males producing the majority of leader signals in 39% of all trials (figure 8*a*). In all of these trials, these males also exhibited the highest average chirp duration of all chorus members. However, in 85% of trials females approached ‘frequent leaders’ defined as males producing most or second most leading signals (see examples in figure 8*b*). In 64% of trials in which females selected a frequent leader, they also selected a male with higher average chirp duration compared with non-preferred competitors, whereas only 27% of females preferred frequent leader males producing shorter signals (table 1). Disregarding chirp timing, 62% of females selected males with the highest chirp duration of all competitors, in contrast to only 15% of females approaching males with the shortest chirp duration (figure 8*a*).

**Table 1.** Average chirp duration of males in choruses in which females selected ‘frequent leaders’. (Bold numbers indicate the chirp duration of the selected male. Numbers in italic indicate selected males with long chirp durations.)

chorus	1	2	3	4	5	6	7	8	9	10	11
male 1	182.5	213.6	217.8	194.8	201.5	150.3	**159**.**4**	*177*.*0*	**197**.**0**	166.6	173.7
male 2	217.5	**182**.**7**	205.5	178.9	*228*.*2*	178.3	169.4	140.5	171.6	98.1	167.3
male 3	*212*.*5*	191.2	**223**.**9**	**153**.**2**	195.0	*212*.*3*	165.8	162.2	190.8	**155**.**9**	*214*.*4*
male 4	205.3	163.5	178.9	137.8	218.5	185.9	n.a.	157.5	199.1	185.1	190.3

## 5. Discussion

Based on results of chorus synchrony and female preference functions in two different katydid species, Greenfield & Schul [27] suggested the evolution of chorus synchrony either as the outcome of inter-male cooperation or competition. A communication system in which chorus synchrony emerges from both opposing strategies has not yet been described, although this seeming paradox has been proposed for synchronously flashing fireflies [41]. Our study provides evidence for a cooperative beacon effect in a synchronizing katydid that is probably the outcome of the competition for timing signals as leader, owing to strong selection exerted by females. Here, we discuss causes and consequences of chorus synchrony in *M. elongata* on the basis of three different hypotheses frequently used to explain the evolution of chorus synchrony.

### 5.1 ‘Beacon effect hypothesis’

The small time difference of about 70 ms between leader and follower signals in four male choruses indicates a high degree of signal overlap favouring a beacon effect. Chorus signal amplitudes in the centre of such choruses are about 7 dB higher relative to signals produced by solo singing males. A similar value (6.5 dB) was obtained with simultaneous loudspeaker broadcasts of identical chirps, an increase we would expect when four identical incoherent sound sources are active at the same time. However, shifting two signals relative to others by 70 ms resulted in a strong decrease of signal amplitude. We explain this obvious discrepancy between the signal amplitude increase of real males and playback experiments by the plasticity of signals timed as follower. As already described in Hartbauer *et al.* [42] chirps timed as follower consist of fewer soft syllables and exhibit a slightly higher syllable rate, which leads to a gradual reduction of the duration of signals with increasing time lag between signals timed as leader (figure 5). As a consequence, the final syllables of highest amplitude in the chirp show a high degree of temporal overlap in acoustic interactions. This suggests that the observed beacon effect is the result of synchronous signal production and signal plasticity. A similar kind of signal plasticity has been described for another species of katydid (*N. nebrascensis*) and a cicada (*Calloconophora pinguis*; Hemiptera: Membracidae) where follower signals are shorter, such that both signals typically ended approximately at the same time [4,43]. In the latter species, synchronized substrate-borne group signals have higher amplitudes compared with lone signallers, which may facilitate collective foraging in this leafhopper species.

A significant preference for aggregate signals was found in other acoustically communicating insects [4–6] as well as in synchronously flashing fireflies [41]. However, in contrast to the hypothesis that the beacon effect would significantly increase the attraction of females towards the signals of four male choruses this was not the case. We explain the lack of a preference for the chorus signal in females with differences in the fine structure of amplitude modulation (the syllable pattern) within chirps, as being largely obstructed in the chorus signal (electronic supplementary material, figure S1). While the clear syllable pattern of the solo song indicates a signaller in close proximity, a degraded syllable pattern of the chorus signal may indicate a signal that is transmitted over some distance. Nevertheless, the preference for higher signal amplitudes was confirmed in two-choice experiments between chirps of solo singing males.

A beacon effect probably expands the area in which receivers are able to detect chorus signals [8,15–17]. The simulation of the active space of solo and chorus signals demonstrated a 2.5 times larger active space for a group of four synchronizing males, under ideal conditions for sound transmission (figure 6*b*,*d*). However, assuming a random distribution of females and a rather low detection threshold of 40 dB SPL, the per capita mating chances for males contributing to the beacon effect steadily decrease as chorus size increases (figure 6*c*). This has been the main argument against the cooperative hypothesis [14]. By contrast, simulating a higher detection threshold of 50 dB SPL resulted in almost the same mating advantage in chorus versus single males (figure 6*e*). A higher detection threshold is likely in the natural habitat of *Mecopoda* where after sunset, background noise amounts to more than 60 dB SPL [44–47] and SNRs for signal detection decrease. Nonetheless, equal per capita mating possibilities of males in a chorus and lone singing individuals raises the intriguing question about factors that stabilize chorus synchrony in *M. elongata*.

### 5.2 ‘Rhythm preservation hypothesis’

The ‘rhythm preservation hypothesis’ suggests chorus synchrony as the outcome of a cooperative act among males aiming to keep their signal period attractive for females [18]. *Mecopoda elongata* females significantly preferred a static signal period of 2 s over identical, but randomly timed signals exhibiting the same average signal period (figure 7*a*). Therefore, males in a chorus better produce periodic signals in synchrony in order to avoid variable chirp intervals produced by a group of males with different intrinsic signal periods. This situation parallels the communication system of *N. nebrascensis* where the unusually large amplitude modulation required for verse recognition forces males to synchronize their calls in order to preserve an attractive pattern [19]. A similar mechanism has been suggested to shape signal timing in the firefly *P. carolinus* where flash synchrony reduces visual clutter and increases the likelihood of a female flash response [20].

The preference for males timing their signals as leader in acoustic interactions leads to a selection of males with faster intrinsic signal rates (figure 3*b* [33]), which raises the question about mechanisms counteracting a Fisherian runaway selection of signal rate in *M. elongata*. Surprisingly, *M. elongata* females preferred the shorter signal period only in the choice situation 2.0 s versus 2.5 s, whereas in the choice situation 1.5 s versus 2.0 s both song models were equally attractive (figure 7*a*). Furthermore, females were less motivated to approach any speaker in choice situations in which the CPs of model songs were equal or shorter than 1.5 s (figure 7*b*). This result contrasts other communication systems where males with a higher signal rate or calling effort are usually more attractive for females (e.g. *Ephippiger ephippiger* [48]; *Hyla arborea* [49]). The combination of a preference for a CP and female motivation exerts a stabilizing selection on the species-specific CP of about 2 s. Therefore, CP mainly conveys information about species identity rather than about the quality of a signaller. This is supported by results from a diet study, in which *M. elongata* males in poor condition were able to signal as fast as males set on a high quality diet [50]. Similarly, leader males in the frog *H. arborea* did not necessarily exhibit a better body condition, but invested more in sexual displays compared with males of poor condition [49].

### 5.3 ‘Inter-male competition hypothesis’

If synchrony in *M. elongata* choruses results from inter-male cooperation aiming to maintain a species-specific signal rate and increased peak signal amplitude one would expect intrinsically faster signalling males to reduce their signal rate in a chorus relative to solo singing. Contrary to this assumption, males producing a higher number of leading signals in a chorus slightly increased their signal rates. Together with a strong leader preference of females (figure 7*a*), this result suggests chorus synchrony in *M. elongata* to be the outcome of ongoing competition between males aiming to time their signals as leader (see also [25,51]). A preference for signals slightly timed ahead of others is common in many chorusing insect and anuran species [22,52–60]. A time lag of only 13 ms was already sufficient to bias female choice in the synchronizing katydid *N. spiza* [25]. However, the advantage of timing signals as leader in *Mecopoda* can be compensated by increasing the amplitude of follower signals by about 4–11 dB (see also [22,24,28]). Yet, females released among males in small choruses more frequently approached males with leader signals and chirps with higher duration (figure 8). Signal duration plays also an important role for mate choice in the katydids *Ephippiger diurnus* and *E. ephippiger* where leadership is equally important [48] and has the potential to overrule the preference for longer calls and faster rhythms [28]. As signal amplitude was not recorded in our experiments, we cannot exclude that signal amplitude differences between males influenced the choice of females as well. A positive correlation between leading call probability and SPL exists in an Indian *Mecopoda* species [61], which further increases the attractiveness of males timing their signals as leader.

### 5.4 What stabilizes the follower role?

The establishment of consistent leader–follower roles in small *M. elongata* choruses corroborates results obtained in male duets where the intrinsic signal period was identified as a key factor for the likelihood of timing signals as leader [33]. Such persistence of leader and follower roles contrasts findings in other species such as *N. spiza*, *E. diurnus* and *O. fultoni* where the production of leading signals shifted among chorus members [18,25,28]. In an Indian *Mecopoda* species, males singing as followers in one night produced leader chirps in the subsequent night [61]. Males singing as followers in this *Mecopoda* species make use of two different strategies to compensate their apparent disadvantage arising from signal timing: strategic spacing and singing when leaders are quiet. Up to now such strategic spacing has not been studied in the *Mecopoda* species of this study. As already discussed in [42], a recently discovered tachinid fly parasitoid homing in on *Mecopoda* males may represent a strong disadvantage for males signalling as leaders, because a related fly species *Ormia ochracea* exhibits a similar leader preference in a choice situation [62]. Thus, there is the intriguing possibility that *Mecopoda* males suffer from a trade-off when signalling as leaders, being preferred by conspecific females, but at the same time have a higher risk of parasitation, owing to a convergent preference between *Mecopoda* females and fly parasitoids. In this way, the combination of sexual and natural selection may lead to opposing selective forces acting on the individual, which relaxes the competition for timing signals as leader. Furthermore, males in a chorus probably benefit from a slightly increased mate attraction, which is not necessarily the case for eavesdropping predators [30]. However, this hypothesis has to be tested in future choice tests with the fly paraitoids.

### 5.5 Evolution of chorus synchrony in *Mecopoda elongata*

Multi-level selection theory [63–65] explains cooperation among non-related individuals on the basis of selective factors acting on a group of individuals. Our study revealed sexual selection as the major driving force that favours synchronous chorusing in a *Mecopoda* species because females select males frequently producing leading signals as well as chirps of longer duration in a chorus. As a consequence, males are forced to compete for timing signals as leader which in turn results in a rather strong beacon effect as a by-product of inter-male competition. Contrary to our expectation, even a rather strong amplitude increase of group signals did not result in a significantly higher attractiveness of small choruses over solo singing males. This would suggest inter-male competition rather than cooperation to be responsible for the evolution of imperfect chorus synchrony. In a multi-agent simulation study, Nityananda & Balakrishnan [66] demonstrated that synchrony is likely to evolve if females prefer leading signals as well as group signals of higher amplitude. However, their simulation was based on a much stronger beacon effect than observed in our measurements, and thus appears less appropriate for simulating the evolution of chorus synchrony in the *Mecopoda* species of this study. Therefore, the question remains what prevents the evolution of alternative signalling strategies that may appear in the form of lone singing males, non-synchronizing males (non-resetters), males avoiding the production of signals timed as follower (e.g. *N. spiza* [25]) and mute males intercepting females originally attracted to leaders. In the game-theoretical model of Kokko [67], female preference for larger leks favours aggregate formation, whereas within-group choosiness of females simultaneously limits the size of evolutionary stable leks as the consequence of inter-male competition. This model applied to *Mecopoda* suggests that males gain a fitness benefit by signalling in small groups, but the resulting beacon effect probably does not explain increased inter-male competition for mates in a chorus. We suggest that other factors like enemy confusion generated by synchronously signalling males may stabilize chorus synchrony in this *Mecopoda* species [29–31].

## Supplementary Material

Figure S1: Oscillogram of the solo and chorus signal. Timing and signal structure of signals used in experiment 3.

## Supplementary Material

Figure S2: Relative change in the CP durations between solo singing males and males singing in a small chorus. Positive values indicate a reduction of CPs in the chorus relative to solo singing; negative values indicate the opposite. Box and whisker plots are based on 13 leaders and 39 followers.

## Data accessibility

Data is available from the Dryad Digital Repository: http://doi.org/10.5061/dryad.q824f.
