# Supplementary figures and images for: Competition and cooperation in a synchronous bushcricket chorus
Source: R Soc Open Sci. 2014 Oct 8;1(2):140167. doi: 10.1098/rsos.140167 (PMC4448899; doi:10.1098/rsos.140167)

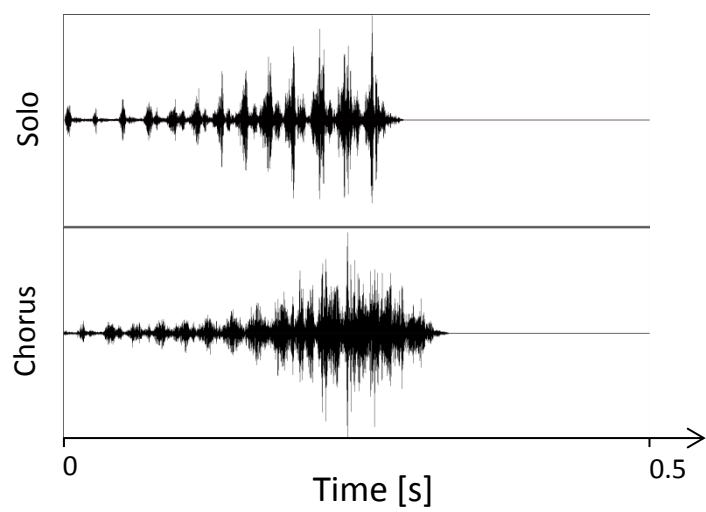

Supplement: Figure S1: Oscillogram of the solo and chorus signal. Timing and signal structure of signals used in experiment 3. [file rsos140167supp1.pdf]

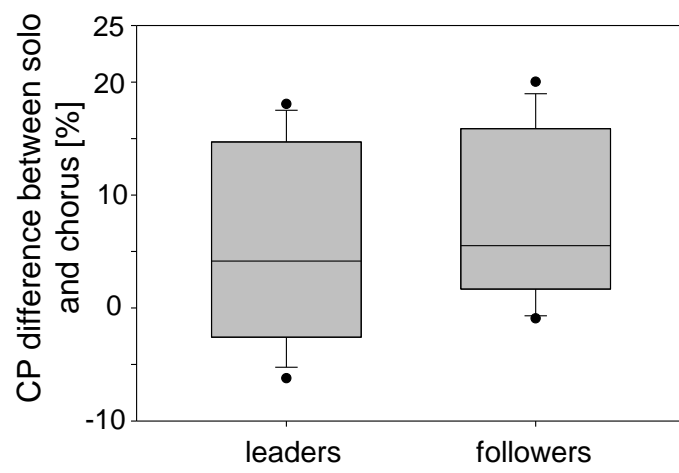

Supplement: Figure S2: Relative change in the CP durations between solo singing males and males singing in a small chorus. Positive values indicate a reduction of CPs in the chorus relative to solo singing; negative values indicate the opposite. Box and whisker plots are based on 13 leaders and 39 followers. [file rsos140167supp2.pdf]
